# Whiskers-less HIV-protease: a possible way for HIV-1 deactivation

**DOI:** 10.1186/1423-0127-20-67

**Published:** 2013-09-12

**Authors:** Mohammad Reza Dayer, Mohammad Saaid Dayer

**Affiliations:** 1 Department of Biology, Faculty of Science, Shahid Chamran University, Ahvaz, Iran; 2 Department of Parasitology and Medical Entomology, Tarbiat Modares University, Tehran, Iran

**Keywords:** HIV-1 protease, Inhibitors, Flap, Whisker truncation

## Abstract

**Background:**

Among viral enzymes, the human HIV-1 protease comprises the most interesting target for drug discovery. There are increasing efforts focused on designing more effective inhibitors for HIV-1 protease in order to prevent viral replication in AIDS patients. The frequent and continuous mutation of HIV-1 protease gene creates a formidable obstacle for enzyme inhibition which could not be overcome by the traditional single drug therapy. Nowadays, *in vitro* and *in silico* studies of protease inhibition constitute an advanced field in biological researches. In this article, we tried to simulate protease-substrate complexes in different states; a native state and states with whiskers deleted from one and two subunits. Molecular dynamic simulations were carried out in a cubic box filled with explicit water at 37°C and in 1atomsphere of pressure.

**Results:**

Our results showed that whisker truncation of protease subunits causes the dimer structure to decrease in compactness, disrupts substrate-binding site interactions and changes in flap status simultaneously.

**Conclusions:**

Based on our findings we claim that whisker truncation even when applied to a single subunit, threats dimer association which probably leads to enzyme inactivation. We may postulate that inserting a gene to express truncated protease inside infected cells can interfere with protease dimerization. The resulted proteases would presumably have a combination of native and truncated subunits in their structures which exert no enzyme activities as evidenced by the present work. Our finding may create a new field of research in HIV gene therapy for protease inhibition, circumventing problems of drug resistance.

## Background

The replication cycle of human immunodeficiency virus type 1 (HIV-1) requires three necessary enzymes. These are reverse transcriptase, integrase and protease. The protease, E.C.3.4.23.16, is an aspartyl protease made of two identical subunits each composed of 99 residues [1-3]. The C-shaped subunits of the protein join each other via non covalent interactions to form a dimeric structure of C2 symmetry. The interactions formed between N and C terminal residues, 1–5 and 95–99 referred to as whiskers, stabilize this dimeric assembly [4,5]. The basic role of the enzyme in HIV-1 infection is the selective and proficient cleavage of peptide bonds in viral glycoproteins of gag and pol genes during viral maturation processes [6-9]. The protease active site involves two catalytic triads composed of “Asp^25^-Thr^26^-Gly^27^” sequence, each from one subunit. Crystallographic studies revealed that the protease active site is covered by a pair of glycine rich, flexible, overlapping loops, extending from residue 43 to residue 58, called ‘flaps’ [10]. Each flap is composed of two anti-parallel beta sheets connected by a beta turn structure. It is proposed that flaps act as a gate to control substrate or inhibitor access to the active site [11-13]. In other word, flaps help in substrate/inhibitor recognition or immobilization of the active site cleft. [14,15]. Being of functional importance, the residues located at the protease active site include about 40 percent hydrophobic residues [10]. These hydrophobic residues enable conformational changes during HIV-1 protease catalysis via exchanging Van der Waals contacts, maintaining structurally significant hydrogen bonds involved in the flap opening [16]. The dissociation of the dimeric enzyme to its subunits results in complete loss of its enzymatic activity. Therefore, the peptide sequence at the contact points of the dimers is highly conserved. It has been shown that complementary structures binding to interface residues prevent protease dimerization but decrease enzyme activity to one third of magnitude [4,17]. However, the low dissociation constant of the enzyme dimers (Kd < 10 nM) indicates the high affinity of monomers to remain in a dimeric association [18-20]. On the other hand, it was shown that truncating only four residues from N terminus of subunits results in formation of monomeric species of extremely decreased activity [20,21]. The whisker truncation removes hydrogen bonds which are normally formed between N and C terminal groups, hence threatening the dimer stability [22].

Given its critical role in viral maturation, the HIV-1 protease is considered to be an important target for drug designing to control HIV consequences [6,13-15,18-20,23]. Inhibitors could be directed to compete with substrates for the enzyme active site or even to interfere with enzyme dimerization. None of these two inhibitory mechanisms are dealt with in the present work, instead, we aimed to simulate three complexes including native, single and double subunit truncated proteins as well as enzyme substrates. The main scope of this work was to study the effect of whisker truncation on the enzyme structure and flap opening status providing details on substrate and enzyme binding site interaction.

## Methods

The crystal structure of wild type protease in complex with a tri peptidyl substrate of Glu-Asp-Leu (PDB ID: 1A30) was used throughout this study. Obtained by X-Ray diffraction method and refined at the resolutions of 2.0 Å, this structure was prepared from protein data bank, (http://www.rcsb.org/pdb) and used as an initial structure for MD simulation [24]. The same structure was used to obtain single truncated (STP) and double truncated (DTP) proteases by deleting four residues from N and C terminals (whiskers) of one or two subunits respectively. Each enzyme-substrate complex was placed in the center of a rectangular box having dimensions of 4.75×6.04×6.24, 4.74×6.03×6.23 or 4.75×6.04×6.24 nm for native, STP and DTP respectively. The boxes were filled with SPCE water molecules using genbox command of gromacs package so that to cover the simulated proteins with water shell of 1.0 nm thickness. Molecular dynamic simulations were performed using double-precision MPI version of GROMACS 3.3.1 installed on UBUNTU version 9.10 with 43A1 force field [25]. The net charges of simulated systems were analyzed by preprocessor engine of GROMACS package. System neutralization was done by adding equivalent number of negative chloride ions. Energy minimization was performed for hydrogen atoms, ions, and water molecules in 1500 steps of energy minimization using steepest descent method to minimize the energy of system to at least 300 kJ/mol. LINCS algorithm was used to apply constraint on bonds lengths. The SETTLE algorithm was used to constrain the geometry of water molecules. The systems were then subjected to a short molecular dynamic with all-bonds restrains for a period of 500 ps before performing a full molecular dynamics without any restrains [26]. Molecular dynamic simulations were carried out for 20 ns at 37°C and 1 atmosphere. Berendsen, Thermostat and Barostat, were used for temperature and pressure coupling respectively and Particle Mesh Ewald (PME) method for electrostatic interactions. The time steps of 1 femtosecond were applied to all simulations. All of these simulations were done at neutral pH (Asp, Glu, Arg, and Lys ionized) [27,28]. The protein binding site residues of protease were extracted using ArgusLab 4.0.1 software (Mark A. Thompson, Planaria Software LLC, Seattle, WA, http://www.arguslab.com) before and after simulations and the energy minimized. The Statistical Package for the Social Science (SPSS-PC, version 15. SPSS, Inc., Chicago, IL) was used to analyze the data. The differences between parameters were considered significant at p < .05.

## Results

**Figure 1:**
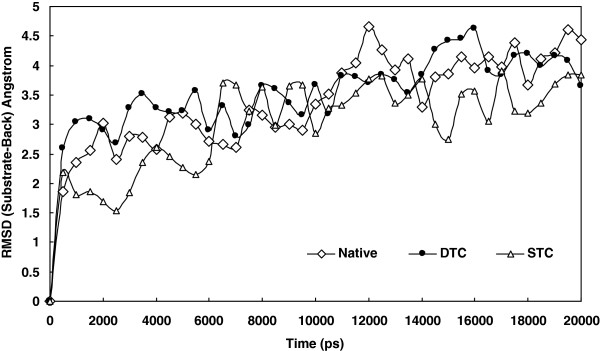
RMSD plot for native, STC and DTC complexes with the protease obtained for 20 ns simulation at 37°C and 1atomsphere in explicit water box.

**Figure 2:**
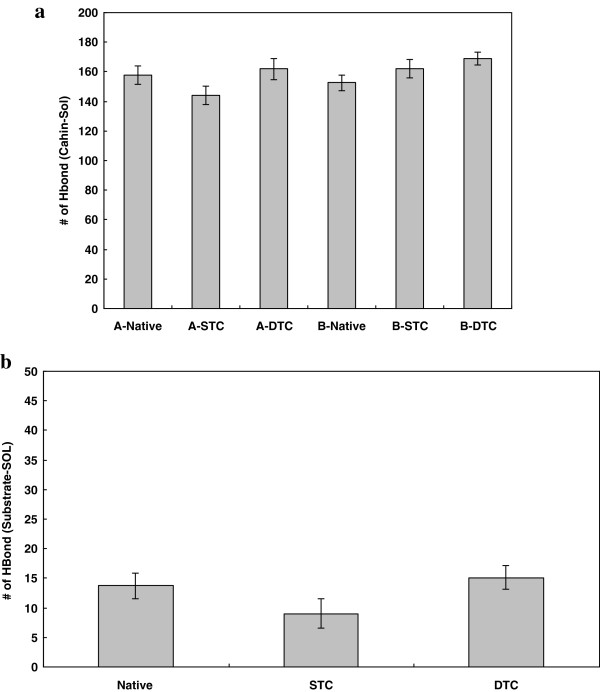
Average number of hydrogen bonds formed, a: intra A and B chains of native, STC and DTC variants of the protease during simulations, b: between the substrate and bulk solvent for native, STC and DTC variants of the protease during 20ns simulations at 37°C and 1atomsphere in explicit water box.

**Figure 3:**
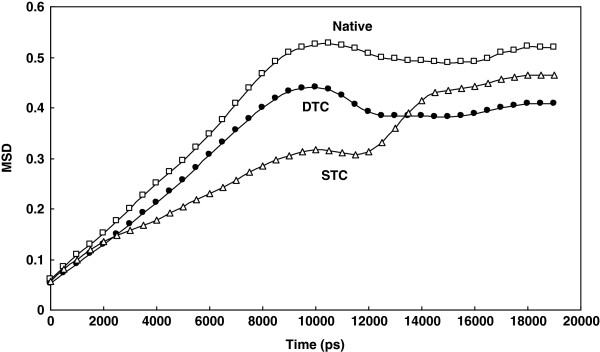
Changes in mean square displacement of the enzyme substrate during simulation for native, STC and DTC complexes (The data obtained from 20 ns simulation at 37°C and 1atomsphere in explicit water box.

**Figure 4:**
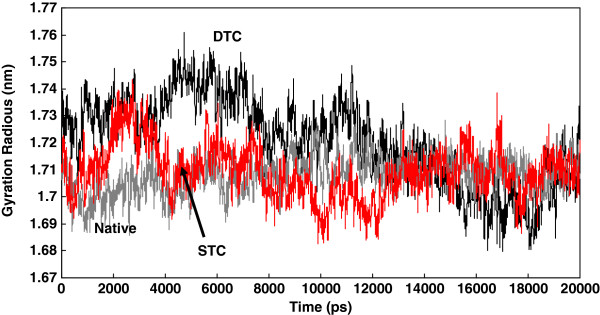
The plot of gyration radius of dimeric protein for native, STC and DTC complexes during 20 ns of simulation (The data obtained from 20 ns simulation at 37°C and 1atomsphere in explicit water box).

**Figure 5:**
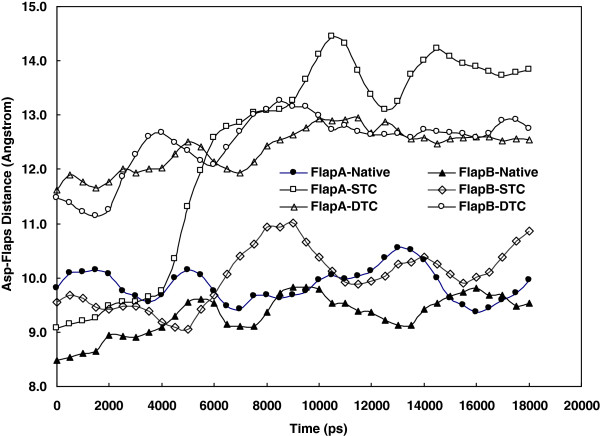
Changes in the distance between Asp25 and Ile50 (Flap distance) during simulation for 20 ns period (The data obtained from simulations trajectories for up to 20 ns simulation period at 37°C and 1atomsphere in explicit water box).

**Figure 6:**
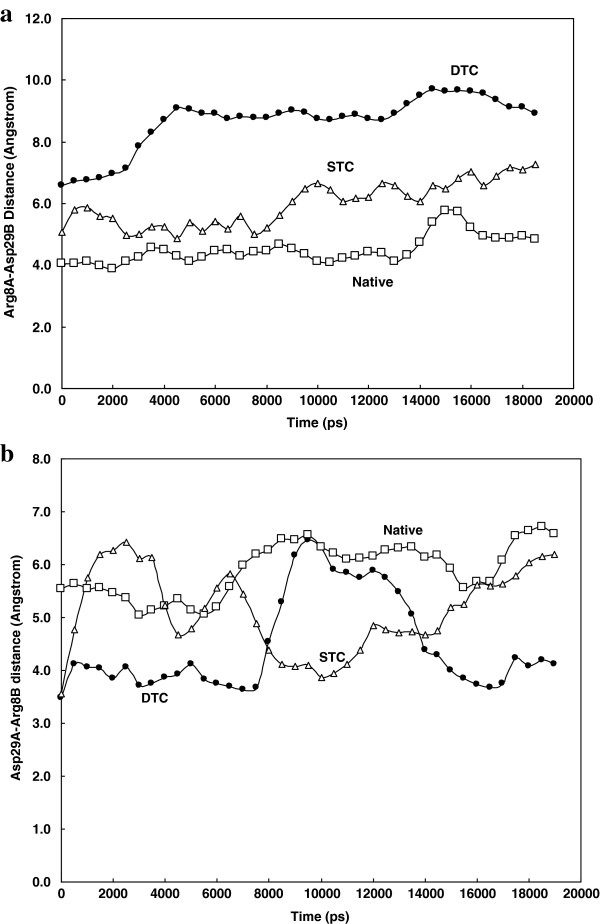
Change in the distance between, a) Arg8 of chain A and Asp29 of chain B during 20 ns simulation period, b) Arg8 of chain B and Asp29 of chain A during 20 ns simulation at 37°C and 1atomsphere in explicit water box.

Figure 1 shows root-mean-squared deviation (RMSD) changes of the substrate compared to the enzyme back-bone during simulation for native, single (STC) and double truncated enzymes (DTC). The similar pattern of progression in RMSD of all three complexes and the states of equilibrium indicates similar structural alterations. The counts of hydrogen bonds formed between each of A and B subunits and the solvent are shown in Figure 2a. The values of hydrogen bond counts are plotted in Figure 2a as Means ± SD. As depicted, hydrogen bonds formed between subunit B and the solvent tend to increase passing from native, through STC to DTC, although the increase is significant (p-value < .05) only in DTC. The apparent decrease in number of hydrogen bonds formed between subunit A and the solvent in STC was because of reduced length of A chain as a result of truncation of four residues from each end. However, comparing hydrogen bonds of native A chain (in native complex) with that of truncated one in DTC indicates that formation of hydrogen bonds is different in both A and B chains. This is why hydrogen bonds show no significant alteration between native, STC and DTC complexes. In general, Figure 2a shows that HIV-1 protease truncation increases protein hydration in its dimeric form. This means that upon truncating residues from N and C terminal, protease becomes more hydrated. For more complication, Figure 2b shows the counts of hydrogen bonds formed between substrate and bulk solution in three complexes (Mean ± SD). As indicated, the substrate in DTC unlike in STC complex is freed to make relatively more hydrogen bonds with water upon protein truncation, while its hydration does not differ significantly from that of the native complex. Figure 3 shows Mean Square Displacement (MSD) progression plot for the substrate during simulation for STC and DTC. It is obvious that in the native complex, the substrate interacts more easily and effectively with the enzyme binding site residues than truncated complexes, and penetrates the protein structure prominently. In contrast, STC and DTC (lower MSD curves) exhibit less diffusing properties. This finding indicates that upon protein truncation, the binding of substrate to enzyme results in a weak and less active protein. The apparent biphasic behavior of STC in the range of 8000-14000 ps *is an indication of the asymmetric nature of STC complex which prevents free movement of the substrate inside the enzyme active site as could be revealed by such a small simulation period as 14000 ps. Nonetheless, the expected higher MSD for STC compared with that for DTC may be reachable beyond 14000 ps.* Figure 4 shows the gyration radius of protein changes during simulation. As indicated, the gyration radius is significantly higher for STC and DTC than the native structure. The distance between Asp^25^ and Ile^50^ from the same subunit have been reported as an index of flap opening or closing of the protease. This distance in subunit A and subunit B is measured during simulation using g_dist command of gromacs and plotted in Figure 5. The single or double truncated enzyme sequence increases this distance meaningfully and leads to flap opening of the binding site. More precise examination of the protein structure reveals that there are two cavities opened to the enzyme active site. The first cavity is placed in the front side of the protease, giving the enzyme a three dimensional structure with two flaps, two ears, nose and whisker on the front side [22]. There is a salt bridge formed between Arg^8^ from subunit A and Asp^29^ from subunit B and positioned to the outer edge of this cavity. The changes in the distance between Arg^8^A and Asp^29^B could be used as a measure of opening or closing of this gate during simulation. Figure 6a shows that Arg^8^A-Asp^29^B distance increases upon truncation of N and C terminal residues. The second cavity is placed on the opposite or back side of the protease. The outer edge of this cavity is lined by a salt bridge formed between Asp29 from subunit A and Arg8 from subunit B (Asp^29^A-Arg^8^B distance).

Figure 6b shows Arg^8^B-Asp^29^A distance for the native and truncated systems. As shown, there was no significant change in this distance indicating that probably no alteration took place in gating status of this cavity.

## Discussion

The human immunodeficiency disease caused by HIV-1 virus is amongst the most threatening diseases which affect the physical and psychological well being of human societies. The fast mutating behavior of HIV genome which overtakes the progress of anti HIV drug discovery constitutes the main driving force of more experimental and theoretical research on viral proteins. The main scope of this study was to find critical hints that may help in shutting down the viral infection. We have, therefore, used a coordinate file of wild type protease in complex with a tri peptidyl substrate (Glu-Asp-Leu) as a native complex throughout the experiments. A single subunit truncated complex was prepared by cutting four residues from N and C terminal. A double truncated complex (DTC) was also prepared by omitting the same residues from both subunits. In order to capture enough snapshots from molecular events caused by protease truncations, including system equilibration, substrate-binding site interactions, flap opening and dimerization status, all simulations were carried out for 20 ns duration. Figure 1 presents RMSD change of native, STC and DTC complexes during simulations. The resulted curves confirm that all three complexes encountered the same structural alterations so that all RMSDs changed from 1.5 to about 4 Angstroms, which have, otherwise, variance not exceeding 0.5 Angstrom. These two characteristics of RMSD curves allowed comparative analysis of the result of complex simulations and the trajectory file. Being an indicator for conformational status, the hydrogen bonding of the protease subunit and substrate with solvent is highly sensitive to structural alteration e.g. changes in dimer compactness. As depicted in Figure 2a, the mutual stabilization of subunits A and B with solvent through hydrogen bonding increases significantly upon whisker truncation. Also, Figure 2b indicates that the hydrogen bonds formed between the substrate and solvent increase upon similar whisker truncation. Figure 2a and 2b together confirm that whisker truncation is responsible for reduced enzyme activity as previously reported [14,17], probably because of its association with loosely joined monomers in dimer assemblies and weakly attached substrates to the enzyme binding site, Mean square displacement (MSD) of the substrate is a useful parameter for determining the substrate penetration inside the enzyme structure during simulation. The elevated MSD curve is an indication of the presence of a more freely diffusing substrate, while reduced MSD progression curve indicates a trapped substrate in the structural cavity with local minimum energy. As appeared in Figure 3, the whisker truncation decreases MSD in a proportional manner i.e. MSD is higher in the native complex than in STC which, in turn, exhibited MSD value higher than DTC at above 14000 ps of simulation*.* As expected, the protein radius increased upon dimer dissociation. The gyration radius obtained by g_gyrate command in gromacs package could be used as the protein average radius. As shown in Figure 4, Rg of truncated complexes was significantly higher than that of the native complex at the beginning of simulation, but it slowly decreased to reach an equilibrium value equal to that of the native structure. Nevertheless, the protease tertiary structure seems to be globular, although this is not true from the side view. In fact, the globular shape of the protease resembles a biconcave disc similar to that of red blood cells. Therefore, the decrease in Rg during simulation can be a result of a simple rotation of the enzyme molecule around perpendicular axis, instead of being caused by extensive changes in dimer compaction. Thence, the reduction of gyration radius may not be attributed to changes of the protein radius but to the rotation of molecule during simulation. For a meaningful comparison of whisker truncations, the gyration radius of all complexes was calculated in a situation where all molecules had similar spatial orientation exclusively at 5 ns simulation. Figure 4 indicates that the highest Rg belongs to doubly truncated whiskers associated with the least compacted structure. The increase or decrease in distance between Asp^25^ and Ile^50^ of a given subunit (Asp^25^-Ile^50^ distance) is already introduced as a criterion for flap opening or closing respectively [29]. A distance of more than 15.8 Angstrom is considered to represent a semi opened conformation for the flap gate [29]. It is also shown that flaps open during ligands (substrate or inhibitor) entry or exit and they remain closed in case of ligands settlement in catalytic site. Figure 5 plots Asp^25^-Ile^50^ distance for each subunit separately against time. This figure confirms statistically that all substrate-enzyme systems demonstrate closed conformations with Asp^25^-Ile^50^ distance of less than 15 Angstroms. However, in whisker truncated complexes there are notable increases in Asp^25^-Ile^50^ distance from 9 to about 15 Angstroms in STC and from 10 to 13 Angstrom for DTC. The results presented in Figures 2, 3 and 4 confirm that whisker truncations loosen the dimeric structure and convert it to a structure of lower compactness. Figure 5, however, indicates that whisker truncations not only decrease dimer compaction but also increase flap opening detectable by Asp^25^-Ile^50^ distance measurement. As far as the STC complexes are concerned, this distance is the same as in native complexes at the beginning of simulation. This means that truncating a single whisker causes no detectable alteration in Asp^25^-Ile^50^ distance after a short period of simulation. However, after a longer simulation of 4 ns or more, the effect of STC on flap opening can be detected. Although flap opening or closure measured by Asp^25^-Ile^50^ distance has been used by researchers to explain substrate and enzyme interactions, this cannot provide satisfactory interpretation for some molecular events which demand more sophisticated and detailed explanations. As shown earlier in the *Results* section, we introduced another valuable index namely the distance between Arg^8^A-Asp^29^ of B subunit. Figure 6a shows that whiskers truncations cause a slight movement of A subunit far from B subunit during simulation. This displacement enlarges the size of front cavity that opens toward the protease binding site and facilitates the gating mechanism mediated by flaps. Figure 6b shows that, from backside view, the distance between Asp^29^A-Agr^8^ of B subunit remains in the same position in the protease. As illustrated, this distance shows no remarkable differences for our three complexes. This finding provides evidence that upon whisker truncation, the dimer opens more prominently from front side than from back side. This may lead to active site opening and probably enzyme deactivation.

## Conclusions

Based on our findings we can conclude that upon whisker truncation in one or two subunits, the protease undergoes vast structural alterations which yield open configuration of the binding site, hence rendering the enzyme to be catalytically inactive as shown by other authors [22]. Also, we postulate that gene therapy via inserting HIV-protease gene truncated at whiskers domains into infected cells may open up a new horizon in HIV treatment. Whisker less protease presumably interferes with dimerization of the viral protease and consequently leads to production of mixed type proteases with no (or less) enzymatic activity.

## Abbreviations

MD: Molecular dynamics; STC: Single truncated protease; DTP: Double truncated protease; RMSD: Root mean square displacement; MSD: Mean square displacement; Rg: Gyration radius.

## Competing interests

Both authors declare that they have no competing interests.

## Authors’ contributions

Mohammad Reza Dayer developed the concept and designed and performed all experiments and analyzed the obtained results and built the main idea of the work. Mohammad Saaid Dayer, however, participated in overall discussion of the work and in English editing. Both authors read and approved the final manuscript.
